# Glances at the Hospitals

**Published:** 1903-08-08

**Authors:** 


### GLANCES AT THE HOSPITALS.

### SURREY COUNTY HOSPITAL, GUILDFORD.

The ordinary income reached £5,625, and the ordinary expenditure £5,491, showing a small balance in favour of the hospital at the end of the year's work. The funds have received several additions during the year. Mr. Hartmann gave £500, Mr. Baring-Gould £100, Mr. Shepherd £100, an anonymous donor £23, and Mr. Williamson has presented ground rents of the value of £9 8s. per annum. At the beginning of the year the hospital contained 71 patients. Of the total number discharged 595 were cured, 125 relieved, 31 not relieved, 46 died, and 55 remained under treatment. The average daily number resident was 66, and the average cost per week was £1 9s. 4d. The medical and surgical tables merely give numbers of patients who were treated for various diseases. Without a statement of results these tables are almost useless.

### THE DARLINGTON HOSPITAL.

The ordinary income amounted to £2,500, and the ordinary expenditure to £2,545. There was a balance in the treasurer's hands, over from last year, of £138. The work-people's subscriptions have fallen off slightly, being £1,114, instead of £1,137 for the previous year. The average number of patients in residence was 45, the average cost per patient per month was £4 4s., and the average residence of each patient was 30 days. The hospital is being enlarged at a cost of £12,000. On January 1st the hospital contained 42 patients, and 509 were admitted during the year, making a total of 551 under treatment. Of these 408 were discharged cured, 55 were discharged relieved, 10 for other reasons, 36 died, and 42 remained in the hospital. The death-rate was 6·5 per cent., but 6 died within 24 hours of admission. The average residence of each patient was 30 days. The medical and surgical tables are divided into 6 columns, showing respectively the total number—the cured, the relieved, the deaths, the unrelieved, and the untreated. Of 5 cases of pneumonia all were cured, and of 7 cases of broncho-pneumonia 4 were cured, 1 relieved, and 2 died. Seeing that the ordinary medical and surgical cases are so carefully tabulated, it is the more curious that the operation table states no results. We notice that there were 5 operations for appendicular abscess, and 7 for removal of the appendix, but we are not informed how many of these were successful. Trephining of the mastoid process was done 5 times, and it would have been interesting to know the results. At least 301 operations were performed. No anæsthetic table is given.

